# Editorial: Natural products as drivers in drug development for neurodegenerative disorders, volume II

**DOI:** 10.3389/fphar.2023.1329769

**Published:** 2023-11-17

**Authors:** Joana Silva, Rebeca Alvariño, Márcia I. Goettert, Hector J. Caruncho, Celso Alves

**Affiliations:** ^1^ MARE—Marine and Environmental Sciences Centre/ARNET, ESTM (School of Tourism and Maritime Technology), Polytechnic University of Leiria, Peniche, Portugal; ^2^ Department of Pharmacology, Faculty of Veterinary, University of Santiago de Compostela, Lugo, Spain; ^3^ Department of Pharmaceutical and Medicinal Chemistry, Institute of Pharmacy, Eberhard Karls Universität Tübingen, Tübingen, Germany; ^4^ Division of Medical Sciences, University of Victoria, Victoria, BC, Canada

**Keywords:** natural products, neuroinflammation, oxidative stress, mechanisms of action, neuropharmacology, drug discovery, ethnopharmacology

The increasing prevalence of neurodegenerative disorders (NDs) poses a huge challenge to public health, partly due to extensions in average life expectancy but also to the need for preventive diagnostics and effective disease-modifying treatments (Logroscino et al., 2022). As per the World Health Organization (WHO), this upward trend is expected to persist into the 21st century. By 2050, it is projected that the elderly population (aged >60 years) will surpass the number of young individuals (aged <15 years). Furthermore, the WHO anticipates that in the near future, neurodegenerative disorders will surpass cancer as the second most common cause of death, with cardiovascular diseases retaining their position as the most common cause (Silva et al., 2023). Neurodegenerative disorders, such as Alzheimer’s Disease (AD), Parkinson’s Disease (PD), Multiple Sclerosis (MS), and amyotrophic lateral sclerosis (ALS), encompass a diverse group of chronic pathological conditions characterized by the gradual loss of the structure and functions of the central nervous system or peripheral nervous system (Rehman et al., 2023). Several biological events, including abnormal protein dynamics, oxidative stress, mitochondrial dysfunction, impaired bioenergetics, dysfunction of neurotrophins, and neuroinflammation, have been related to ND development (Jellinger, 2010; Rehman et al., 2023). Despite significant progress in our understanding of the pathogenesis of NDs, most of the medicines available on the market are not effective and primarily serve to relieve symptoms (Lamptey et al., 2022). Advances in the knowledge about the etiology of NDs and the development of innovative therapeutic agents for their treatment are crucial.

Nature has played a crucial role over the centuries as a source of therapeutic options for application in a wide range of pathologies, inspiring the development of new drugs for emerging threats to human health and reducing disease spread and mortality (Dzobo, 2022). Diverse bioactive compounds with complex structures, novel pharmacological properties, and distinct mechanisms of action have been discovered in plants, animals, microbes, and marine organisms (Najmi et al., 2022). To date, distinct chemical classes of natural products have been reported, including alcohols, alkaloids, amino acid derivatives, aromatic compounds, polysaccharides, fatty acids, lactones, peptides, polyacetylenes, polyketides, quinones, quinolones, sphingolipids, sterols, terpenes, and terpenoids, among others (Newman and Cragg, 2020). Using synthetic chemistry and combinatorial chemistry, new derivatives can also be prepared to develop more effective drugs (Najmi et al., 2022). Opposed to traditional medicine, which utilizes whole plant extracts or a mixture of natural products for treatment, modern scientific practice requires the isolation and assessment of individual compounds from these extracts to determine their potential as pharmaceutical agents. However, due to the pathophysiology complexity of NDs and the ability of mixtures and/or extracts of natural products to interact with multiple simultaneous biological targets, their potential to provide cognitive benefits has been explored (Chen et al., 2021). The innate functions of bioactive natural products to interact with biological targets (e.g., proteins, enzymes, nucleic acids, etc.) with great specificity and affinity can be useful in new drug discovery and development, as well as in therapeutic target identification challenges (Suresh et al., 2023).

The Research Topic presented here addressed the potential of natural products (NPs) as scaffolds for the development of new drugs for the treatment of neurodegenerative disorders, emphasizing the diversity of molecular targets and mechanistic effects. This Research Topic gathered a collection of two critical reviews and two original research articles, with over 8,000 views and more than 2,000 article downloads, highlighting the capacity of NPs to mediate beneficial effects on distinct neurological disorders.

Huang et al. provided a critical review regarding the potential applications of silk fibroin in the development of drug-delivery materials to treat neurological disorders. This natural polymer reveals excellent biocompatibility and a wide range of applications for end-use material formats, including microsphere, nanoparticles, gel, coating/film, scaffold/conduit, and microneedle, and enables the dynamic release of loaded drugs for desired therapeutic responses. At the cellular level, silk fibroin was displayed to protect neuronal cells, stimulate their regeneration, and improve memory deficits by attenuating ROS production in ischemic injury. In traumatic brain injury, SF hydrogels transporting drugs were able to cross the blood-brain barrier, inhibiting glial proliferation and stimulating vascular regeneration.

Substantial evidence suggests a connection between systemic vascular conditions and the neurodegenerative processes that occur prior to cognitive decline and dementia (Kalaria, 2012; Kapasi and Schneider, 2016). Studies conducted within the community setting indicate that individuals with a prior history of stroke have a post-stroke dementia prevalence of approximately 30% (Leys et al., 2005). Chen and Jin reported a detailed review concerning the complex network of signaling pathways and mechanisms involved in stroke pathophysiology. The authors also discussed the potential of traditional Chinese medicine (TCM) therapies such as Rehmanniae and Astragalus to treat stroke consequences due to their multi-target and multi-pathway mechanisms of action.

Wu et al. reported the clinical benefits of Yuan-Zhi Decoction (YZD), a traditional Chinese medical formulation, in the treatment of Alzheimer’s disease (AD). The use of liquid chromatography coupled with mass spectrometry identified 27 unique chemical components, which were demonstrated by network pharmacology and molecular docking analysis to interact with 34 potential molecular targets involved in 26 biochemical pathways. The *in vivo* treatment with YZD improved the spatial orientation and memory of animals with the AD phenotype. At the cellular level, these effects were accompanied by a decrease of the Aβ plaques and tau protein deposition in the hippocampi region. A decrease of BACE1 and β-amyloid levels was also observed, as well as downregulation and upregulation of p-GSK-3β/GSK-3β and p-AKT/AKT pathways, respectively.

Kang et al. studied the neuroprotective activities of oligosaccharides extracted from Rehmanniae Radix [RR, the dried tuberous roots of *Rehmannia glutinosa* (Gaertn.) DC.], which is used in traditional Chinese medicine. Their extraction was performed from different starting materials, namely, dried Rehmannia Radix (ODRR) and prepared Rehmannia Radix (OPRR). The samples were tested on an *in vivo* model of AD, *Caenorhabditis elegans*, leading to a delay of paralysis, improved learning ability, and prolonging the lifespan of the nematode worm. The mechanism behind these effects seemed to be related to a decrease in reactive oxygen species levels, aggregation, and toxicity of Aβ through the modulation of gene expression of several factors related to these biological events.

Altogether, the four publications gathered in this Research Topic provide a relevant overview of natural products derived from traditional Chinese medicine and natural polymers as potential therapeutic neuroprotective agents in different neurological disorders, including AD, ischemic injuries, and traumatic brain injury.
